# Metabolic adaptation to consume butyrate under prolonged resource exhaustion

**DOI:** 10.1371/journal.pgen.1010812

**Published:** 2023-06-22

**Authors:** Sophia Katz, Claudia Grajeda-Iglesias, Bella Agranovich, Alia Ghrayeb, Ifat Abramovich, Sabrin Hilau, Eyal Gottlieb, Ruth Hershberg

**Affiliations:** 1 Rachel & Menachem Mendelovitch Evolutionary Processes of Mutation & Natural Selection Research Laboratory, Department of Genetics and Developmental Biology, the Ruth and Bruce Rappaport Faculty of Medicine, Technion-Israel Institute of Technology, Haifa, Israel; 2 Department of Cell Biology and Cancer Research, the Ruth and Bruce Rappaport Faculty of Medicine, Technion-Israel Institute of Technology, Bat Galim, Haifa, Israel; 3 Department of Cancer Biology, The University of Texas MD Anderson Cancer Center, Houston TX, 77056, USA; Uppsala University, SWEDEN

## Abstract

Bacteria must often survive following the exhaustion of their external growth resources. Fitting with this need, many bacterial species that cannot sporulate, can enter a state known as long term stationary phase (LTSP) in which they can persist for years within spent media. Several recent studies have revealed the dynamics of genetic adaptation of *Escherichia coli* under LTSP. Yet, the metabolic consequences of such genetic adaptation were not addressed. Here, we characterized the metabolic changes LTSP populations experience, over the first 32 days under LTSP. This allowed us to link genetic adaptations observed in a convergent manner across LTSP populations back to their metabolic adaptive effect. Specifically, we demonstrate that through the acquisition of mutations combinations in specific sets of metabolic genes, *E*. *coli* acquires the ability to consume the short chain fatty acid butyrate. Intriguingly, this fatty acid is not initially present within the rich media we used in this study. Instead, it is *E*. *coli* itself that produces butyrate during its initial growth within fresh rich media. The mutations that enable butyrate consumption allow *E*. *coli* to grow on butyrate. However, the clones carrying these mutations rapidly decrease in frequency, once the butyrate is consumed, likely reflecting an associated cost to fitness. Yet despite this, *E*. *coli* populations show a remarkable capability of maintaining these genotypes at low frequency, as standing variation. This in turn allows them to more rapidly re-adapt to consume butyrate, once it again becomes available to them.

## Author summary

Bacteria are able to survive prolonged periods of resource exhaustion. This ability is crucial for survival within many natural environments. Here, we link the metabolic changes the model bacterium *Escherichia coli* experiences during the first 32 days of growth under resource exhaustion to its genetic adaptation. We show that *E*. *coli* produces an important short chain fatty acid, butyrate, during the first 24 hours of growth within rich media. *E*. *coli* is not initially able to consume this metabolite but adapts to do so during our experiments. We show that these genetic adaptations enable growth on butyrate, but that the clones carrying them likely suffer a cost, when no butyrate is available. Despite these costs, and despite our populations being well mixed and subject to strong selection, these mutations are maintained at low frequencies within the evolving populations. This enables these populations to more rapidly re-adapt to consume butyrate. Our findings likely exemplify the more general ability of bacterial populations to maintain previous metabolic adaptations as standing variation, allowing rapid re-adaptation to previously met conditions.

## Introduction

The ability of bacteria to survive within nutrient limited environments has fascinated the scientific community for many decades [1]. This ability results from bacterial evolution in natural environments, where bacteria are likely repeatedly exposed to short periods of feast and long periods of famine. Bacteria pursue several strategies to face long periods of starvation. Some bacterial species can undergo sporulation, allowing them to maintain complete or near complete dormancy for many years. While sporulation has rightfully received much attention, most known bacterial species cannot sporulate, yet many can still survive for years following the exhaustion of their nutrients, by entering a state termed long term stationary phase (LTSP) [2,3]. In LTSP a sub-population of cells can survive by recycling the remains of their deceased brethren and ancestors.

When bacteria, such as the model bacterium *Escherichia coli*, that are able to enter LTSP are placed in fresh media, they undergo a short period of lag, in which they adjust to growth, and then experience a short period of exponential growth. Following exponential growth, they enter a rather brief stationary phase, in which the number of viable cells remains fairly constant. However, *E*. *coli* cannot maintain itself for very long in stationary phase, and thus rather rapidly enters death phase, a period in which population viability decreases rapidly. A small minority of cells is able to survive death phase and enter LTSP. It is important to note that despite the use of the term ‘stationary’, LTSP is a phase of growth that is entirely distinct from stationary phase, and that cells can be far from stationary under LTSP.

We and others have previously reported on evolutionary experiments designed to probe the dynamics of *E*. *coli* genetic adaptation under LTSP [4–9]. In one such experiment, initiated in July 2015 and ongoing until this day, we established five independent populations by inoculating *E*. *coli* cells into rich media and incubating them within a shaking incubator. ~10 clones from each of these five populations were sequenced, at nine time points, spanning the first three years of the experiments. Results of these experiments thus far show that *E*. *coli* LTSP populations rapidly adapt genetically, in an extremely convergent manner, through mutations to several important regulatory and metabolic genes [4,5].

The first adaptation we observed to occur in *E*. *coli* under LTSP, involves mutations within highly specific sites of the RNA polymerase core enzyme (RNAPC) [4]. A second, highly convergent adaptation that emerges with great temporal precision involves mutations to fatty acid metabolism genes. Specifically, at day 22 of our experiments we observed that, across independently evolving populations, ~80% of sequenced clones carry, in addition to the RNAPC adaptation, a mutation in the *fadR* gene which encodes a master regulator of fatty acid metabolism [10], combined with a second mutation in either *atoC* or *atoS*, that together encode a two-component system that regulates fatty acid metabolism [11]. The adaptations within *fadR* and *atoC*/*atoS* that appear with high convergence at day 22 were not seen at the previous (day 11) or subsequent (day 32) sampled time points [4].

The ability of bacteria to enter LTSP and persist in the absence of external nutrients for many years constitutes a metabolic mystery. Yet, while some focus was given to studying metabolism during stationary phase [12], the metabolic particulars of LTSP have not been addressed. Here we provide the first characterization of the metabolites present within LTSP exhausted media, over the first 32 days under LTSP. This in turn allowed us to ascertain the function of the *fadR* + *atoC*/*atoS* adaptations, demonstrating that these enable the consumption of the short chain fatty acid butyrate, which we show *E*. *coli* produces during the first 24 hours of growth in rich media. We could further show that despite the clones carrying these adaptations suffering an apparent cost, and despite our populations being well mixed, *E*. *coli* LTSP populations are able to maintain minorities of cells carrying these adaptations, even when no butyrate is present. Such maintenance allows populations that adapted to consume butyrate, to re-adapt to consuming this metabolite much more rapidly than populations that never encountered butyrate before.

## Results

### Characterization of the metabolites present within LTSP media as a function of time spent under LTSP

**Fig 1:**
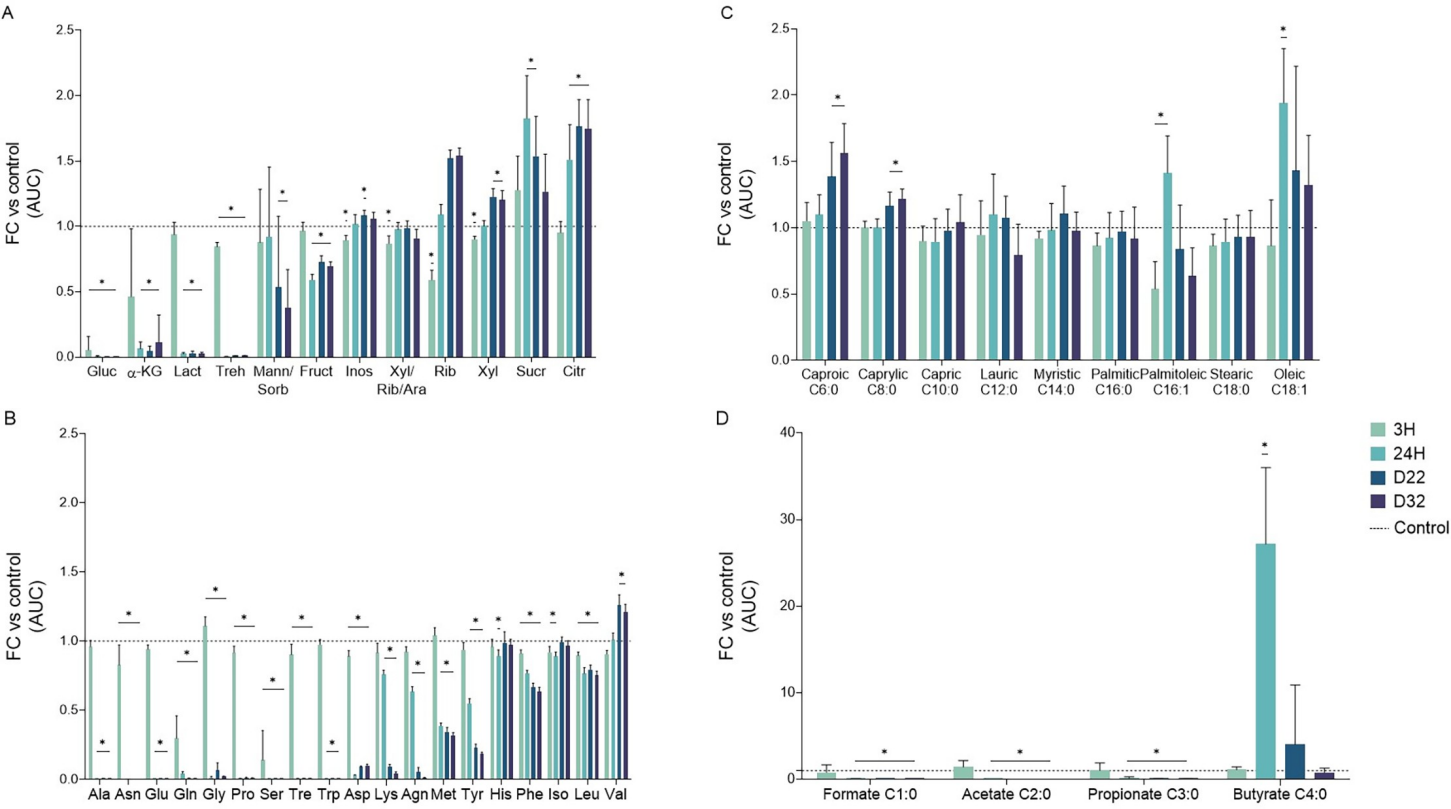
Changes in the presence of metabolites within LTSP media, during the first 32 days of adaptation. Data obtained from five to nine independent LTSP populations, which were established as described in the text. For each population, consumed and excreted metabolites were identified in the media, following 3 h, 24 h, 22 days and 32 days of culture. Six flasks containing only LB without bacteria were used as controls. The fold change (FC) area under the curve (AUC), compared to the controls, was calculated for each identified metabolite at the indicated time point. (A) Glucose and other sugars and derivatives, (B) proteogenic amino acids, and (C) medium- and long-chain free fatty acids were analyzed by targeted LC-MS. (D) Short-chain fatty acids were identified by targeted GC-MS, previous derivatization. Mean values with error bars representing standard deviations around the mean are shown. Statistical significance between two samples was calculated using a Mann-Whitney two-sided test, to compare the absolute values from each timepoint versus control. * = P <0.05. (The detailed data and their statistical analyses can be found in **S1–S3 Tables**).

To examine the metabolic consequences of LTSP adaptations, we initiated new LTSP experiments and measured changes of different metabolites in the media over 32 days (Materials and Methods). Sugars serving as main carbon sources such as glucose and its disaccharide trehalose (a major sugar component of LB [13]) were rapidly consumed, following three and 24 hours of growth respectively (**Fig 1A and S1 Table**). In contrast, uptake of fructose, and of sugar alcohols, considered to be metabolic products [14], was minimal even following 32 days. A significant increase in the concentration of the pentoses, ribose and xylose was observed towards day 32, likely due to the breakdown of dying cells and to the deficient utilization of these sugars, relative to other available carbon sources [15,16]. Amino acids, that are relatively not bioenergetically costly to produce, such as glycine, serine, and threonine, were rapidly consumed. In contrast, very little, if any uptake occurred for phenylalanine, histidine, and the branched-chain amino acids leucine, isoleucine and valine (**Fig 1B and S2 Table**), all of which are much more costly to produce [17]. This observation fits well with previous data suggesting a compromise between the cost and the potential energetic benefit of degrading amino acids [18].

### *E*. *coli* populations produce butyrate during initial growth in fresh rich media and adapt to consume it through the acquisition of mutations within *fadR* and *atoC*/*atoS*

**Fig 2:**
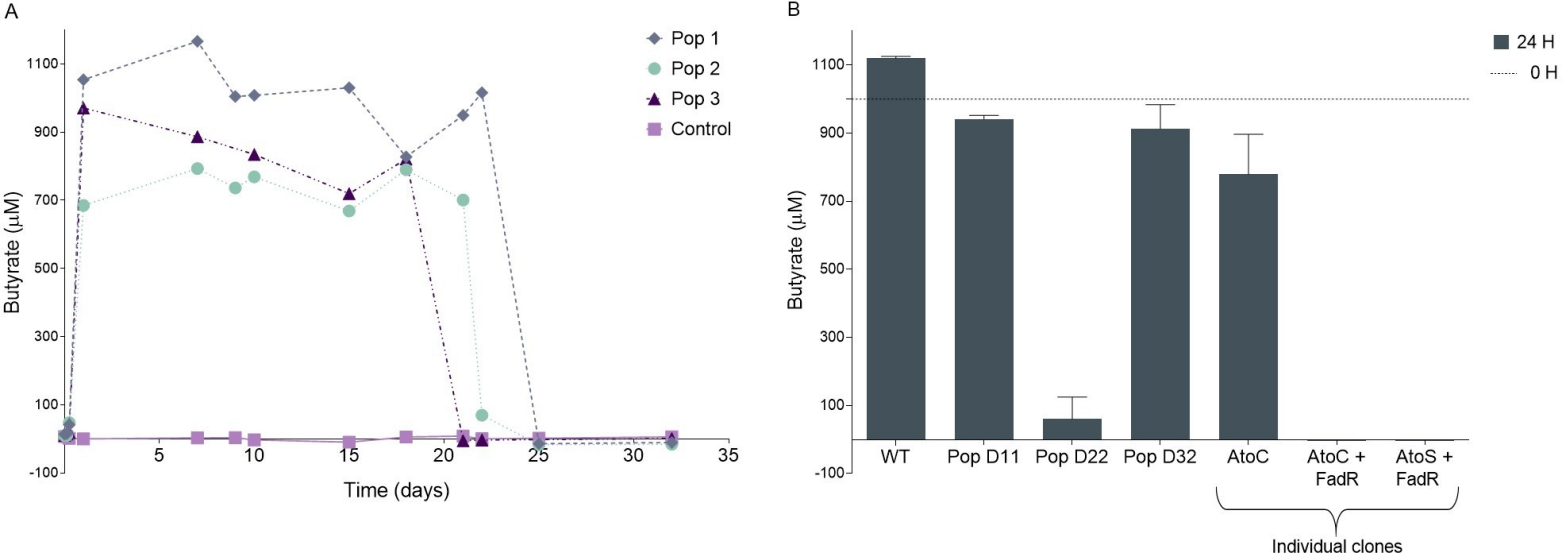
Butyrate kinetics. **(A) Butyrate is produced during the first 24 hours of growth in LB and then consumed at days 18–22.** Butyrate kinetics were monitored for 32 days by GCMS analyses on three independent LTSP populations, with LB media without inoculated bacteria serving as a control (purple). **(B) day 22 LTSP population samples and clones carrying *fadR* + *atoC* or *fadR* + *atoS* mutation combinations can consume butyrate.** The ability to consume butyrate was examined for each sample or clone, through GCMS measurements of the levels of butyrate remaining after 24 hours. Mean values across three biological replicas, per sample or clone, are presented with error bars representing standard deviations around the mean. The levels of butyrate added at day 0 are marked with a dotted line.

The concentration of medium- and long-chain free fatty acids did not significantly reduce with time (**Fig 1C and S3 Table**). However, the short chain fatty acids (SCFA), formate (C1), acetate (C2) and propionate (C3), were consumed by 24 hours (**Fig 1D and S3 Table**). Intriguingly, The SCFA butyrate (C4) was massively produced during the first 24 hours of growth and was then consumed following 18–22 days of culture (**Fig 2A**). Wildtype *E*. *coli* is not known to produce butyrate [19] or to consume it [20]. Accordingly, the ancestral *E*. *coli* K12 MG1655 strain, used to initiate our experiments, is not able to consume butyrate (**Fig 2B**). However, our experiments with LTSP evolved clones, carrying the *fadR* + *atoC*/*atoS* mutations demonstrated their acquired ability to consume butyrate (**Fig 2B**). Population samples from day 22, in which these butyrate consuming clones are present at high frequencies can also consume butyrate, while samples extracted from day 11 and day 32 of LTSP cannot (**Fig 2B**). Taken together, these results suggest that LTSP populations rapidly adapt to consume the butyrate they produced during the first day of growth and that they do so through the convergent acquisition of a specific combination of mutations. Once butyrate is fully consumed, the *fadR* + *atoC*/*atoS* mutations are no longer useful and they undergo sharp reductions in frequencies by day 32 of our original experiment [4]. That this reduction in frequency occurred so rapidly, across multiple populations, suggests that in the absence of butyrate, the clones that carry these mutations suffer a relative cost.

### Butyrate consumption adaptations provide a growth advantage when butyrate is provided, but are rapidly outcompeted once butyrate is no longer available

**Fig 3:**
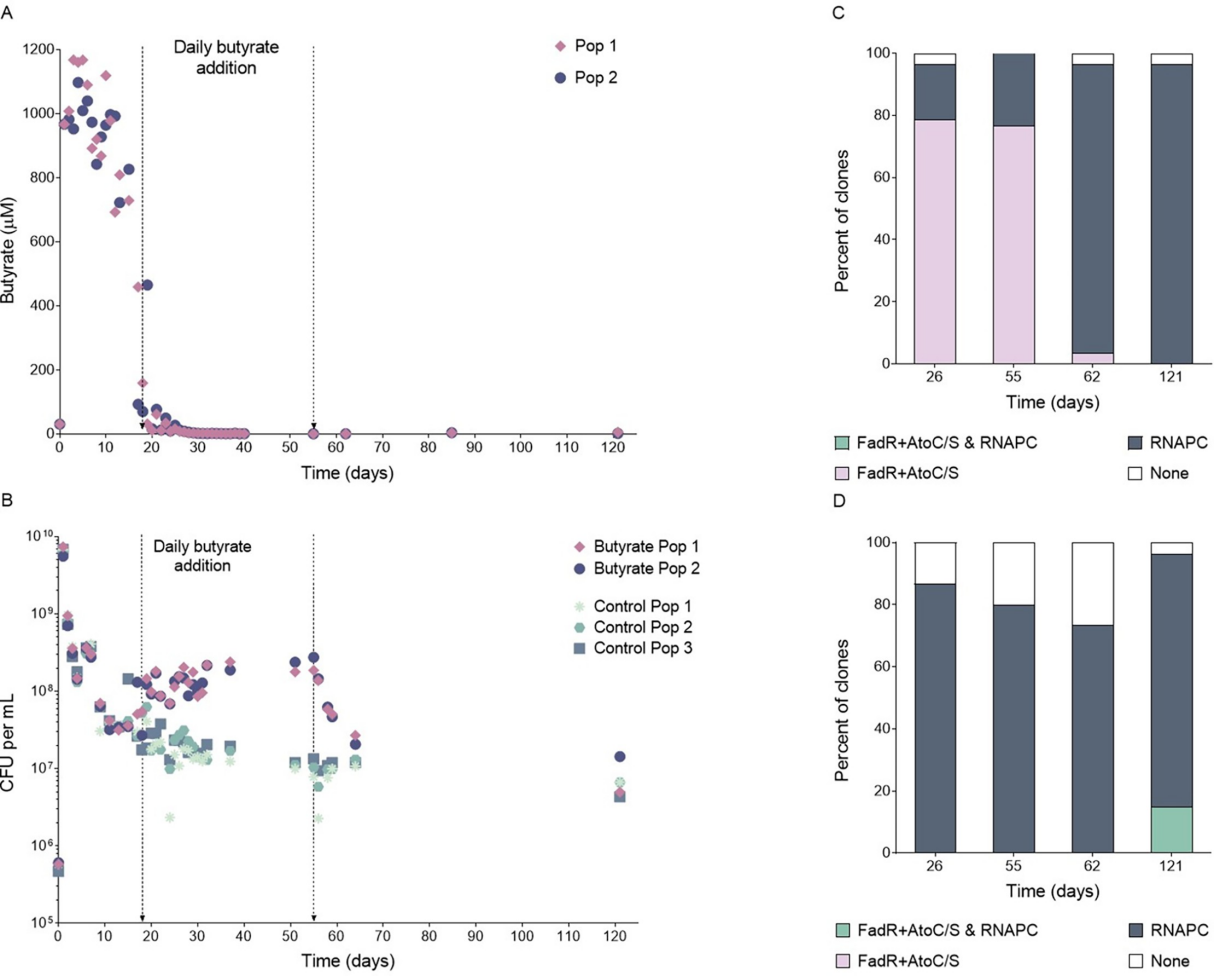
Daily supplementation of butyrate and its effect on LTSP populations. Six LTSP populations were established. To three of the six populations, butyrate was added daily starting at day 18 and ending at day 55. **(A)** Levels of butyrate measured within two populations to which butyrate was added, as a function of time. The time range in which butyrate was added is marked on the graph (A similar plot for the third population is presented in **S1B Fig**). **(B)** Mean number of viable cells with time in the two LTSP populations supplemented daily with butyrate (pink/purple) or in control LTSP populations which were allowed to evolve without interference (green). Numbers of viable cells were estimated by counting colony-forming units (CFU). The time range in which butyrate was added is marked on the graph. (A similar plot for the third population is presented in **S1C Fig**). **(C)** In the three populations to which butyrate is added between days 18 and 55, a high frequency of clones sequenced at day 26 and 55 carry butyrate consumption adaptations but do not carry any adaptations within the RNAPC. Clones sequenced at day 62 very rarely carry butyrate consumption adaptations and often carry an RNAPC adaptation. **(D)** Control populations to which butyrate was not added almost never contain butyrate consumption adaptations at observable frequencies. In contrast, these populations contain high frequencies of clones carrying RNAPC adaptations at all time points.

Next, we sought to examine what would happen if butyrate was continuously available. We established six new LTSP populations. For three of them we supplemented the media daily, from day 18 to day 55, with 1 mM butyrate (a concentration similar to that produced during the first 24 hours of growth (**Fig 2A**)). The remaining three populations served as controls and were allowed to evolve without further intervention. In two of the three butyrate-supplemented populations, the butyrate produced during the first 24 hours of growth was consumed at days 17 and 18 of the experiment, and the supplemented butyrate was then continuously consumed daily (**Fig 3A**). Starting at day 18 and up to day 55, bacteria from these two butyrate-supplemented populations were able to grow substantially more than in the absence of butyrate, reflected in much higher colony forming unit (CFU) counts (**Fig 3B**). In the third population, butyrate produced during the first 24 hours of growth was consumed earlier, at day seven, and butyrate supplemented starting at day 18, initially accumulated, and was only consumed from day 26 onwards (**S1B Fig**). In line with this, the increase in CFU was observed for this population only from day 26 (**S1C Fig**). Combined, these results demonstrate that LTSP bacteria that adapt to consume butyrate can grow on butyrate when it is continuously made available.

We fully sequenced 9–10 clones from each of the six populations at each of four time points: days 26, 55, 62 and 121. At days 26 and 55, clones carrying the *fadR* + *atoC*/*atoS* mutation combination were seen at high frequencies (ranging from 66.7% to 100%) within all three populations supplemented with butyrate (**Figs 3C**, **S1D and S4 Table**). Strikingly, in each of the three populations, more than a single genotype carrying these mutation combinations appeared to segregate simultaneously (**S1D Fig**), highlighting the remarkably extensive genetic variation present within LTSP populations. In contrast, no butyrate consumption genotypes were observed at days 26 or 55 within the control populations (**Figs 3D**, **S1E and S4 Table**). In our original experiments, the *fadR* + *atoC*/*atoS* mutation combination was found within day 22 clones that also carried an RNAPC adaptation [4]. However, all clones sequenced from our butyrate-supplemented populations carried either the *fadR* + *atoC*/*atoS* mutations, or an RNAPC adaptation or, in rarer cases, neither of the two (**Fig 3C** and **S4 Table**). We have previously shown that LTSP RNAPC adaptations severely reduce growth rates in rich media, where nutritional resources are readily available [4,8]. This may explain why these RNAPC adaptations would be disfavored, in butyrate supplemented LTSP populations, in clones that have adapted to be able to grow on butyrate. Yet, clones that carry an RNAPC adaptation, but do not carry mutations enabling them to consume butyrate still persist within our populations at sufficiently high frequencies to allow their detection through the sequencing of a handful of clones out of ~400*10^7^ cells present within our populations. This suggests that these clones either occupy a niche that provides them with an advantage despite their inability to consume butyrate, or that they are maintained at high frequencies for more than five weeks, despite carrying substantial costs to fitness. In contrast, once butyrate supplementation stops (at day 55), clones carrying the butyrate consumption adaptations reduce in frequencies rapidly, so by day 62, no such clones were observed in two of the butyrate-supplemented populations and only a single such clone was detected in the third population (**Figs 3C, S1D and S4 Table**). This suggests that clones carrying the butyrate consumption adaptations suffer costs that rapidly drive their frequencies down once butyrate is no longer available.

### Despite apparent costs, LTSP populations are able to maintain a relatively long-lasting standing variation, allowing them to more rapidly re-adapt to consume butyrate

**Fig 4:**
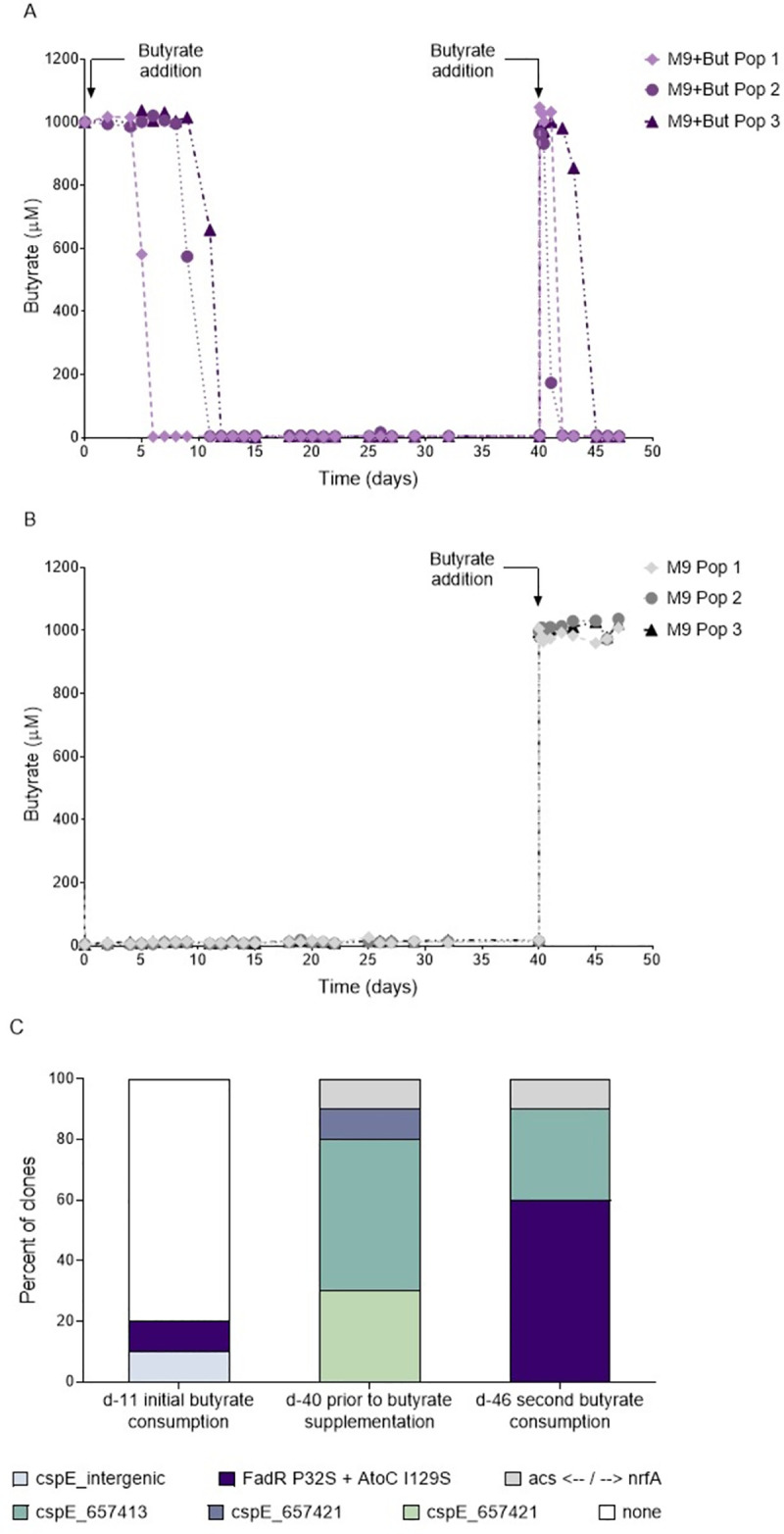
Populations that previously encountered butyrate adapt more rapidly to consume it, once it again becomes available. One day after initiation, cells from three LTSP experiments were filtered out of their LB media and re-inoculated into three flasks of M9 minimal media, either supplemented with 1 mM butyrate **(A)**, or not **(B)**. When butyrate was provided, it was initially consumed by day 12. Following four additional weeks, populations were sampled and assayed for their ability to consume butyrate. As shown, only populations that initially received butyrate could then consume it, during the week following its addition into their media (**A**, arrows represent time points in which butyrate was added). **(C)** Sequencing data from butyrate-supplemented population 3 reveals that the same mutation combination was responsible for initial (day 11) and secondary (day 46) butyrate consumption. Yet, this mutation combination was not present at observable frequencies at day 40, immediately prior to the second supplementation with butyrate. The full list of mutations identified can be found in **S5 Table**.

Bacteria often exist within fluctuating environments, in which a metabolite that became available once, is more likely to become available again later. Being able to rapidly re-adapt to consume a metabolite that a population already adapted to consume once before, may thus be beneficial. Yet, as seems to be the case for the clones carrying the butyrate-consumption adaptations, adapting to consume a new metabolite may often come at a cost to fitness. We wanted to understand whether despite these costs, populations are able to maintain their previous adaptations to consume butyrate as standing variation, enabling them to more rapidly re-adapt to consume butyrate. In other words, we aimed to examine whether populations that once grew in the presence of butyrate and adapted to consume it, would re-adapt more rapidly to consume it, once it was again available. To test this, we needed to obtain control populations that were never exposed to butyrate. Towards this end, we established six new LTSP populations, as usual starting by growing cells in LB. After 24 hours of growth, we filtered the bacteria out of their media, washed and re-inoculated them into M9 minimal media (that contains no butyrate and no other carbon sources). To three of the six populations we added butyrate at similar concentrations to those produced by *E*. *coli* during the first 24 hours of growth in LB (1 mM). Butyrate levels were monitored for all six populations. As expected, no butyrate was detected for the control populations. The M9 populations to which butyrate was added, consumed it earlier than most LB-maintained populations, at day six for one of the M9 populations and day 11 for the remaining two (**Fig 4A**). Once butyrate was consumed from these three populations, we waited an additional four weeks and then sampled each of the six populations and added butyrate to each of the samples, either once (**Fig 4**), or daily for seven days (**S2 Fig**). As shown in **Figs 4B and S2B**, the populations that never saw butyrate, could not consume it for the seven days examined. In contrast, the populations that had seen butyrate and had adapted to consume it, re-adapted to consume it rapidly, doing so within two to five days (**Figs 4A** and **S2A**).

Focusing on the populations that initially received butyrate and then received it again only once, we sequenced 9–10 clones from each population at the time point in which they consumed the butyrate for the second time. In two of the three populations 33.3% to 60% of the clones sequenced carried a *fadR* + *atoC*/*atoS* mutation combination (**S5 Table**). Furthermore, in one of these two populations we could also see the exact same mutation combination at the time at which that population initially consumed the butyrate (day 11 of the experiment (**Fig 4C** and **S5 Table**)). Clones carrying the butyrate-consumption genotype were not observed when sequencing 10 clones from the sampling time-point, prior to the addition of butyrate (**Fig 4C and S5 Table**). Combined, this demonstrates that the genotypes that arise in frequency to consume the butyrate initially, decrease in frequency, so they can no longer be observed four weeks later. However, these genotypes are still maintained within their populations at lower frequency and can thus again increase in frequency to consume the butyrate the second time it becomes available. No mutations of note were identified when sequencing ~10 clones from the other populations at the time they initially consumed the butyrate (**S5 Table**). For the third population we did not observe any clones carrying the *fadR* + *atoC*/*atoS* mutation combination, even once they consumed the butyrate for the second time.

Combined, our results here demonstrate that despite the apparent costs suffered by clones carrying the butyrate-consumption adaptations, they remain within our populations for at least four weeks, following the consumption of butyrate. This in turn allows the populations that previously adapted to consume butyrate to more rapidly re-adapt to consume butyrate, once it again becomes available.

## Discussion

Wildtype *E*. *coli* cannot consume the short chain fatty acid butyrate. Here, we show that *E*. *coli* produces butyrate during the first 24 hours of growth in fresh LB. This butyrate remains within the media until, in a remarkably convergent manner, LTSP clones adapt to consume it, through the acquisition of mutation combinations within the *fadR* and *atoC*/*atoS* genes. These adaptations allow *E*. *coli* to grow on butyrate, when it is daily supplemented into the LTSP media. However, once supplementation stops and butyrate is no longer available, the frequency of the butyrate consumption genotypes convergently reduces within a very short period of time, strongly suggesting that the clones that carry these adaptations suffer a cost. Despite this apparent cost, and despite the fact that our populations are well mixed, and that previous studies have demonstrated them to be subject to very strong selection [4,5], butyrate consumption genotypes remain at lower frequencies within our populations for a substantial period of time. This in turn, allows these populations to re-adapt more rapidly to consume butyrate, once it again becomes available, compared to populations that never encountered butyrate before, and never adapted to consume it.

The apparent costs suffered by the butyrate consuming clones may stem from the butyrate consumption genotypes themselves. However, these costs may also stem from other mutations that are present or absent from these clones. For example, in the experiment in which we continuously added butyrate, during the time butyrate was added, we observed two types of clones: One type adapted to consume butyrate by acquiring the butyrate consumption adaptations, while the second type carried mutations within the RNAPC genes *rpoB* and *rpoC* (**Fig 3C**), which our previous data suggest are strongly adaptive under LTSP [4,5]. Once butyrate was no longer available, the clones carrying the butyrate consumption adaptations rapidly reduced in frequency and the clones carrying the RNAPC adaptations took over the populations. The costs that led to the convergent reduction in the frequency of the butyrate consuming clones could stem directly from the effect of the butyrate consumption mutations or from the absence of RNAPC mutations, or from a combination of both. In the experiments in which we do not add butyrate, the clones that acquire a butyrate-consumption mutation combination, do so on the background of an existing RNAPC mutation [4,5]. There as well, we see convergent rapid reductions in the frequencies of the clones carrying butyrate consumption adaptations, once they consume all the butyrate, that was initially produced during the first day of growth in LB. In this case the absence of RNAPC mutations cannot explain these rapid reductions in frequencies. However, it is still possible that the costs bared by these clones are not the direct result of them carrying butyrate consumption adaptations, but rather the result of the presence or absence of other mutations. It is important to note that no matter the exact cause of the costs suffered by the butyrate-consuming clones, the fact that they appear to remain within the population as standing variation, despite these costs, is remarkable.

A limitation of our study is that for each population and timepoint we sequenced, we sequenced only ~10 clones, out of a population of ~10^8^. This means that we expect to observe only genotypes segregating at very high frequencies and do not expect to be able to detect any mutations that are segregating at slightly lower frequencies. Our inability to always identify clones carrying the *fadR* + *atoC*/*atoS* mutation combination, even when butyrate is being consumed, may be explained by this. A second limitation of our study relates to the experiments in which we demonstrated that M9 LTSP populations are able maintain butyrate consumption adaptations for four weeks following butyrate depletion. The reason we chose to carry out these experiments in M9 is that we needed to obtain control populations, in which butyrate was never available. Such control populations cannot be obtained in LB, because the bacteria themselves produce butyrate during their first 24 hours of growth. However, it is important to note that the dynamics of adaptation within M9 may differ from those that occur in exhausted LB media.

It has long been understood that environmental heterogeneity can support the long-term maintenance of different genotypes, each of which is specialized to a different set of conditions (reviewed in [21,22]). However, within well mixed populations, of the type studied here, there should be no environmental heterogeneity, and conditions can only vary temporally. In such populations, the co-existence of specialist genotypes is thought to be more limited, and possible only under specific sets of circumstances [22–25]. Our results indicate that even for such well-mixed populations genotypes specialized towards previously encountered conditions may be maintained for substantial periods of time. It is possible that our experimental design that, similar to most natural environments, does not dilute out slow growing or dormant cells, enables this maintenance. While most evolutionary experiments rely on continuous culturing or on serial dilution (reviewed in [22,26,27]), which do not allow variation in growth rates to freely persist within the studied populations, our experiment does not artificially limit such variation [5]. Dormant spores or bacterial cells that enter a dormant or extremely slow growing state are at least partially immune from selection [28–30], allowing them to survive despite carrying deleterious genotypes. Dormancy or extreme slow growth is thus one manner in which populations may be able to maintain within them non-favored genotypes, specialized towards a previously encountered condition [31,32]. Balancing selection can also enhance the co-existence of specialist genotypes [33,34]. Enabling populations to maintain higher variation in growth rate may increase the opportunities for such balancing selection [35,36] and may therefore enhance the ability of populations to maintain within them longer-term genetic variation, including genotypes adapted to previous conditions.

One surprising finding of our study is that *E*. *coli* is able to produce butyrate, during its initial growth within fresh LB. Butyrate is possibly the most well-known short-chain fatty acid (SCFA), involved in human body homeostasis. Butyrate serves as the main energy source for colonocytes [37]. Yet, despite its importance to homeostasis, the human body cannot synthesize butyrate and relies on anaerobic fermentation by commensal gastrointestinal bacteria, of the phyla Bacteroidetes and Firmicutes [38]. Butyrate protects the intestinal epithelial integrity and is also involved in apoptosis and in gene regulation [39,40]. Its concentration is about 20mM in the gut lumen, ranges between 14–28 mM in the proximal colon and 4–14 mM in the distal colon (based on its relative molar ratio among SCFA) [39,41]. The main pathway for the production of butyrate is the acetyl-CoA (CoA) pathway which produces butyrate through the degradation of non-digestible carbohydrates. However this pathway is only one of four pathways used to produce butyrate. The alternative pathways (lysine, glutarate, and 4-aminobutyrate) require amino acids as their initial substrate [42]. We were not able to identify the pathway by which *E*. *coli* produces butyrate, however our results (**S3 Fig**) indicate that butyrate is not produced via the acetyl-CoA (CoA) pathway. We therefore speculate that in *E*. *coli* butyrate is probably produced from amino acids. Functional predictions for butyrate producing bacteria identified some anaerobic species of delta class proteobacteria, that are known to consume butyrate, as potential butyrate producers, via the lysine ato pathway [42]. It is possible that the lysine ato pathway is also responsible for *E*. *coli* butyrate production. Supporting this idea are the results of Seregina *et al* that engineered strains of *E*. *coli* to produce butyrate by inducing the expression of the *atoB* gene [43].

We find that *E*. *coli* produces butyrate, despite initially being unable to consume it. It is possible that butyrate is simply a side product with no benefit for *E*. *coli*. However, it is also possibile that butyrate serves as a signaling molecule. Supporting this idea of butyrate as a signaling molecule are its known effects on bacterial gene regulation. In EHEC (enterohemorrhagic *E*. *coli*—a close relative of the strain used in this study that also cannot consume butyrate), low levels of butyrate up regulate virulence factors such as adherence-related genes [41] and in the closely-related *Salmonella typhimurium*, 30mM of butyrate inhibit expression of invasion genes [44]. Since butyrate levels are not constant across the intestinal tract it was suggested that butyrate enables these pathogens to sense their environment for the optimal invasion conditions [41,44]. Also, butyrate is known to modulate the immune system in the gut (reviewed in [40]) and it is therefore possible that *E*. *coli*, as a natural part of the human microbiota, can sometimes benefit by secreting butyrate in order to attune immune responses.

Importantly, our study revealed that the previous metabolic conditions toward which the bacteria are adapted to under LTSP were, in fact, generated by the bacteria themselves. Butyrate is produced and secreted to the media at times of nutritional abundance, and it forms a metabolic pressure selecting for genetic adaptations that enable later butyrate consumption under LTSP. It is therefore plausible that once bacteria are adapted to thrive on a nutritional resource that was initially produced by the same population, they are likely to maintain a type of population level genetic “memory” for such conditions, even if it comes with a cost once the nutrient is fully consumed.

Our results show that large *E*. *coli* LTSP populations can maintain genotypes allowing them to consume a metabolite they previously encountered, as standing variation over relatively long periods of time. This is striking because these populations are well mixed and subject to extremely strong selection [4,5]. When such standing variation is maintained, genetic adaptation to fluctuating conditions can occur at an ecological rather than an evolutionary time scale, as re-adaptation to previously met conditions does not require novel mutations to occur. Establishment of long-lasting standing variation that includes genotypes that enable the consumption of metabolites that are periodically encountered within a certain environment could also enable bacterial populations to obtain a better ‘foot-hold’ within their environment, by offering a type of protection from invasion by outside populations. After all, populations that already established the ability to consume the metabolites which are most commonly available within a particular environment, will have an advantage in their ability to re-adapt more rapidly to consume these metabolites, compared to an entirely naïve external population.

## Materials and methods

### Media preparation

Luria-Bertani (LB) medium was prepared with 10 g L^−1^ tryptone (BD), 5 g L^−1^ yeast extract (BD), and 5 g L^−1^ NaCl. For (LB) agar plates, 15 g L^−1^ agar (BD) were added to the same formulation. Minimal medium (M9) was prepared according to manufacturer instructions (Difco M9 Minimal Salts, 5× Cat. No. 248510), and supplemented with 2 mL L^−1^ MgSO_4_ 1 M, and 0.1 mL L^−1^ CaCl_2_ 1 M. Butyrate stock solution (Sigma Aldrich CAS no. 107-92-6) was prepared in ultra-pure water at a concentration of 100 mM, and added for a final concentration in the culture medium of 1 mM. All media and solutions were either filter sterilized (0.45 μm pore size) or autoclaved before use.

### LTSP experiments

Single colonies of *E*. *coli* K12 MG1655 (WT, ancestor) were grown in test tubes containing 4 mL of fresh LB medium in a shaking incubator (225 rpm at 37°C), until they reached an OD ~ 0.4. Then, 1 mL was taken to a 2 L-polycarbonate breathing flask containing 400 mL of fresh LB, for a final concentration of ~ 2x10^6^ cells/mL. Flasks were placed in an incubator set at 37°C. The populations are well mixed, by constant shaking at 225 rpm, which does not allow for the establishment of spatial structure. No new nutrients or resources were added to the cultures with time, except for sterile water that was added to compensate for evaporation every 10–15 days, according to the weight lost by each flask during that time period.

Samples from the culture media were taken at regular intervals, centrifuged (5 min at 5000*g*/ 4°C), and the supernatant was stored at -20°C for further metabolomics studies. To estimate bacterial viability, 1 mL of each culture was sampled. Dilutions were plated on LB agar plates, using a robotic plater to evaluate viability through live counts. Samples were stored in 50% glycerol at -80°C for future analysis.

For the daily supplementation of butyrate experiment, single colonies were allowed to recover in 4 mL-fresh LB in a shaking incubator (225 rpm at 37°C), until an OD ~ 0.4 was reached. From each of three ancestral clones, two independent LTSP experiments were started (for a total of six experiments), as described, by inoculating *E*. *coli* K12 MG1655 into 400 mL-fresh LB medium, to a final concentration of ~ 2x10^6^ cells/mL. Flasks were kept in a shaking incubator at 37°C 225 rpm. Starting at day 18 of these experiments, three populations (each one from a different ancestor) were supplemented daily with 1 mL of butyrate (for a final concentration of 1mM), up to day 55. For each butyrate-supplemented population, an additional flask with no supplementation was kept as a control. Population sampling was done regularly.

For the M9 experiments, single colonies were allowed to recover in 4 mL-fresh LB in a shaking incubator (225 rpm at 37°C), until an OD ~ 0.4 was reached. From each of three ancestral clones, 2 independent LTSP experiments were started (for a total of six experiments), as described, by inoculating *E*. *coli* K12 MG1655 into 400 mL-fresh LB medium, to a final concentration of ~ 2x10^6^ cells/mL. Flasks were kept in a shaking incubator at 37°C/ 225 rpm during 24 h. Next, the total population in each flask was harvested by centrifugation (20 min at 4000g) and rinsed once in M9. All populations were resuspended in 400 mL of M9, three of them (each one from a different ancestor) received butyrate at a final concentration of 1 mM. At day 40 of the LTSP-M9 experiments, 20 mL of each of the six populations were withdrawn and split into two 50 mL-Erlenmeyer flasks (10 mL per flask). Each flask received either a single or a daily dose of butyrate (1 mM final concentration). The Erlenmeyer cultures were kept in a shaking incubator at 37°C/ 225 rpm, for one week. Culture media samples and CFU measurements were conducted as described above.

### Monitoring butyrate consumption

In order to monitor the consumption of butyrate, Frozen cultures from our previous study [4], of *E*. *coli* K12 MG1655 (WT, ancestor), LTSP populations 1,2 and 4 at days 11, 22 and 32, and isolated colonies from the clones summarized in **S6 Table**, were thawed and allowed to grow overnight in liquid LB. Additionally, the wildtype ancestral strain was also thawed and grown overnight. At the following day, each type of resulting culture was diluted 1:50 into fresh LB media and recovered for an additional two hours, until they reached an OD ~ 0.2. Cells were harvested by centrifugation (5 min at 5000*g*) and rinsed twice in M9 media. The cells were then resuspended in 5 mL of M9 (baseline) or M9 supplemented with butyrate (1mM final concentration). Tubes were placed in a shaking incubator (225 rpm at 37°C) and sampled at time 0, 3 and 24 hours. Culture media samples were obtained after cell centrifugation (5 min at 5000*g*/ 4°C) and stored at -20°C for further metabolomics analysis.

### Profiling short-chain fatty acids (SCFA) by GCMS

Representative metabolite standards were used for quality control. ^13^C-labeled isotopes of formate, propionate, butyrate, and ^2^H_3_-acetate, were used as internal standards for quantification.

### Chemical derivatization for SCFA

The chemical derivatization procedure of samples using ethyl chloroformate (ECF) was adapted from the protocol described in [45]. Briefly, 200 μL of medium sample was added to a 1.5 mL microfuge tube, followed by addition of 10 μL of internal standard, 10 μL of benzyl alcohol, 10 μL of sodium hydroxide 1 M, and 50 μL of pyridine. The tube was then placed on ice for 5 min. Next, 20 μL of ECF were added immediately followed by vigorous vortexing for 30 s. As gas builds up in the microfuge tube during the derivatization reaction, it is very important that samples are cold enough. Tubes may be carefully opened in the middle of the vortexing period to relieve pressure and put back to vortex to complete the required length of time. After vortexing, 200 μL of MTBE were added, the sample vortexed for another 20 s, and centrifuged at 10,000*g* or max speed for 5 min. 120 μL microliters of the resulting upper layer were transferred to a GC vial with insert for analysis.

### SCFA detection and quantification

Derivatized samples were analyzed with an Agilent 7890B GC system coupled to a 7000 Triple Quadrupole GC-MS system. The column was Phenomenex ZB-1701 column (30 m x 0.25 mm x 0.25 μm), with the following oven program: initial temperature 50°C/ hold 2 min; increment at 10°C/min to 140°C/ hold 0 min; increment at 20°C/min to 182°C/ hold 1 min; and increment at 50°C/min to 280°C/ hold 0 min. Run time 16.1 min. Samples (1 μL) were injected using split mode (20:1, 28 mL/min split flow). The column gas flow was held at 1.4 mL/min of He. The temperature of the inlet was 280°C, the interface temperature 230°C, and the quadrupole temperature 150°C. The column was equilibrated for 2 min before each analysis. The mass spectrometer was operated in single-ion monitoring (SIM) mode between 9.9 and 14.0 min. A segment for each compound was defined: benzyl-formate at 9.9 min, fragments *m/z* 136 and 137; benzyl-acetate at 11.0 min, fragments *m/z* 150, 151, 152 and 153; benzyl-propionate at 12.2, fragments *m/z* 164, 165, 166 and 167; and benzyl-butyrate at 13 min, fragments *m/z* 178,179,180, and 182. All segments at 2.5 cycles/s. Agilent MassHunter Workstation Software (version B.07.01 SP1) was employed for automated data processing, using peak area for absolute concentrations calculations.

### Metabolites profile by LCMS

#### Metabolite extraction

For the extraction of secreted metabolites, 50 μL of medium was diluted in 950 μL of ice-cold extraction solvent (50% methanol, 30% acetonitrile, 20% water), vortexed at high speed/ 4°C during 10 min, centrifuged at 16 000*g* for 10 min at 4°C. Supernatant was transferred into LC-MS glass vials and stored at −80°C until measurement. For the extraction of intracellular metabolites cell pellets were vortexed with 200 μL of ice-cold extraction solvent during 10 min, and incubated at -20°C for another 10 min. These cold-vortex / incubating steps were repeated three times. Pellets were subsequently centrifuged at 16000*g* for 10 min at 4°C, and the supernatant were transferred into LC-MS glass vials with inserts and stored at −80°C until measurement.

#### LC-MS measurements

LC-MS metabolomics analysis was performed as described previously [46,47] (Mackay et al., 2015; Meiser et al. 2016). Briefly a Q Exactive Orbitrap mass spectrometer (Thermo Fisher Scientific) was used, together with a Thermo UltiMate 3000 high-performance liquid chromatography (HPLC) system. The HPLC setup consisted of a ZIC-pHILIC column (SeQuant, 150 mm x 2.1 mm; Merck KGaA, 5 mm), with a ZIC-pHILIC guard column (SeQuant, 20 mm x 2.1 mm) and an initial mobile phase of 20% of 20 mM ammonium carbonate (pH 9.2) and 80% acetonitrile. Cell and medium extracts (5 μL) were injected, and metabolites were separated over a 15-min mobile phase gradient, decreasing the acetonitrile content to 20%, at a flow rate of 200 μL/min and a column temperature of 45°C. The total analysis time was 27 min. All metabolites were detected across a mass range of 75 to 1 000 mass/charge ratio (*m/z*) using the Exactive mass spectrometer at a resolution of 35 000 (at 200 *m/z*), with electrospray ionization and polarity switching to enable both positive and negative ions to be determined in the same run. Lock masses were used, and the mass accuracy obtained for all metabolites was below 5 ppm. Data were acquired using Thermo Xcalibur software. The peak areas of different metabolites were determined using Thermo TraceFinder 4.1 software, where metabolites were identified by the exact mass of the singly charged ion and by known retention time on the HPLC column, using an in-house MS library built by running commercial standards of all metabolites detected.

### DNA extraction, sequencing library preparation and mutation calling

Frozen cultures were thawed and dilutions were plated on solid LB-agar plates and grown over night. Colonies to be sequenced were used to inoculate 4 ml of LB medium in a test tube and were grown until they reached an OD of 1. One milliliter of the culture was centrifuged at 5,000 g for 5 min and the pellet was used for DNA extraction. The remainder of each culture was then archived by freezing in 50% of glycerol at -80°C. DNA was extracted using the Macherey-Nagel NucleoSpin 96 Tissue Kit. Library preparation followed the protocol outlined in Baym et al [48]. Sequencing was carried out at Admera Health (New Jersey USA) using an Illumina HiSeq machine. Clones were sequenced using paired end 150 bp reads. In order to call mutations, the reads obtained for each clone were aligned to the *E*. *coli* K12 MG1655 reference genome (accession NC_000913). Mutations were recorded if they appear within a clone’s genome, but not within the ancestral genome. Alignment and mutation calling were carried out using the Breseq platform, which allows for the identification of point mutations, short insertions and deletions, larger deletions, and the creation of new junctions [49].

## Supporting information

S1 Table Metabolomic analysis of sugars. (XLSX)

S2 Table Metabolomic analysis of amino acids. (XLSX)

S3 Table Metabolomic analysis of fatty acids. (XLSX)

S4 Table Mutations found within continually supplemented butyrate and control populations. (XLSX)

S5 Table Mutations found within M9 clones from populations that initially received butyrate. (XLSX)

S6 Table Genotypes of clones tested for their ability to consume butyrate. (XLSX)

S1 Dataset Raw Metabolomics data. (ZIP)

S1 Fig Daily butyrate supplementation and its effect on LTSP populations. **(A)** Levels of butyrate measured for the three control populations, to which butyrate was not artificially supplemented. **(B)** Levels of butyrate for the third butyrate supplemented population **(C)** Mean number of viable cells, as measured through CFU calculations for the third butyrate-supplemented population. As can be seen, as with the remaining two populations (Fig 3A and 3B), for this population as well, CFU increases, once butyrate begins to be consumed. Dashed lines mark the time frame (days 18–55) during which butyrate was daily added to the media. **(D)** The frequency of different genotypes within the three populations to which butyrate was added between days 18 and 55. **(E)** The frequency of different genotypes within the three control populations to which butyrate was not added.
(JPG)

S2 Fig Populations that previously encountered butyrate adapt more rapidly to consume it, once they again encounter butyrate. One day after initiation, cells from three LTSP experiments were filtered out of their LB media and re-inoculated into three flasks of M9 minimal media, either supplemented with 1 mM butyrate **(A)**, or not **(B)**. When butyrate was provided, it was initially consumed by day 12. Following four additional weeks, populations were sampled and assayed for their ability to consume butyrate, which was supplemented daily into the sampled populations. As shown, only populations that initially received butyrate **(A)** could then consume it, during the week following its addition into their media (arrows represent time points in which butyrate was added).
(JPG)

S3 Fig Formate, acetate and propionate, but not butyrate, are produced from glucose consumption. Full labeled glucose (M+6) was added into the M9 minimal media culture. Ancestral *E*. *coli* K12 MG1655 cells were sampled and assayed for their ability to produce butyrate during 24 hours of culture. As shown, only the SCFA formate **(A)**, acetate **(B)** and propionate **(C)** were detected, in their unlabeled (M+0) and fully labeled (M+1, M+2, M+3, respectively) form. Butyrate, labeled or unlabeled, was not detected at any time point. These results indicate that *E*. *coli* does not produce butyrate via the acetyl-CoA (CoA) pathway.
(JPG)
