# Supplementary figures and images for: Metabolic adaptation to consume butyrate under prolonged resource exhaustion
Source: PLoS Genet. 2023 Jun 22;19(6):e1010812. doi: 10.1371/journal.pgen.1010812 (PMC10321620; doi:10.1371/journal.pgen.1010812)

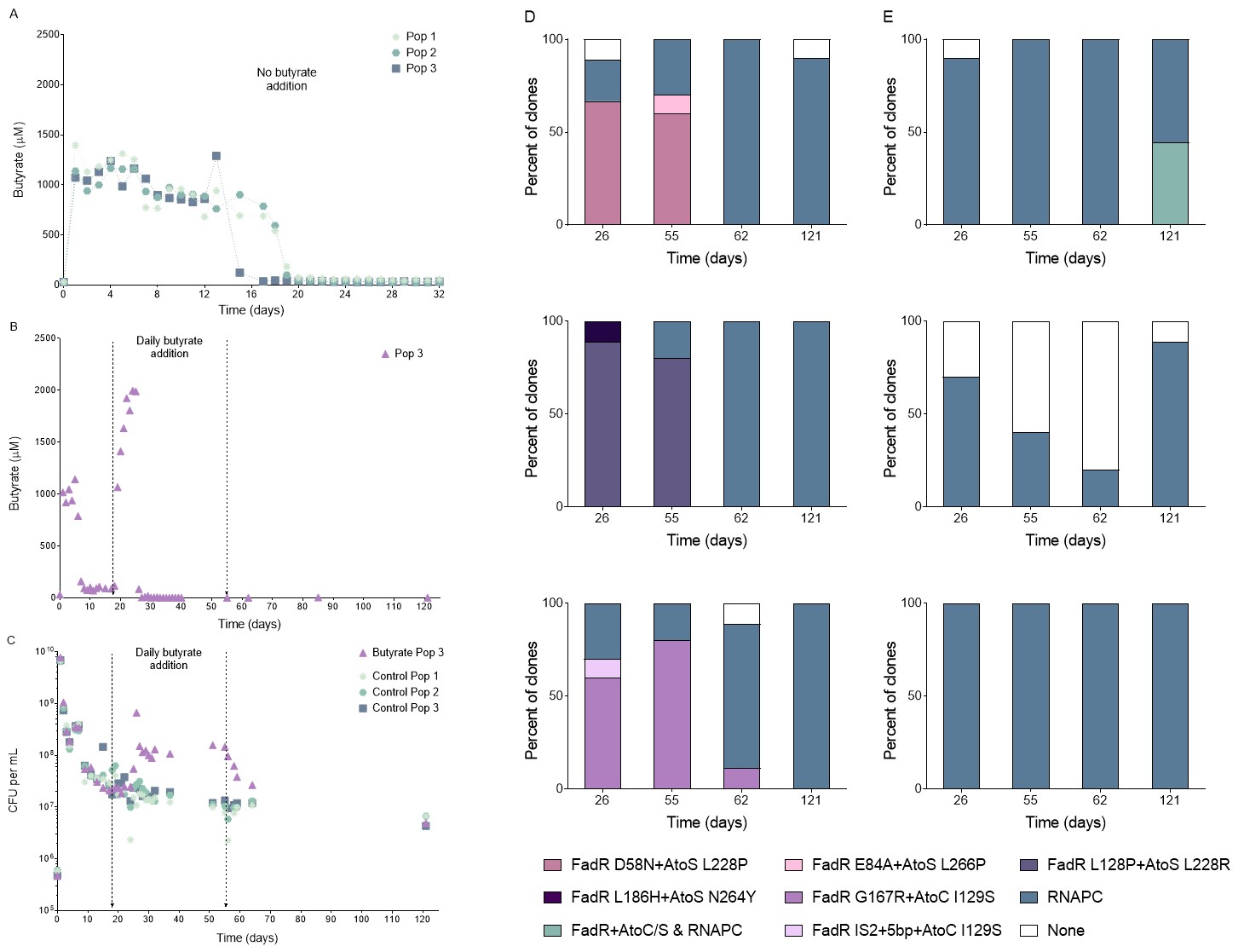

Supplement: S1 Fig — (A) Levels of butyrate measured for the three control populations, to which butyrate was not artificially supplemented. (B) Levels of butyrate for the third butyrate supplemented population (C) Mean number of viable cells, as measured through CFU calculations for the third butyrate-supplemented population. As can be seen, as with the remaining two populations (Fig 3A and 3B), for this population as well, CFU increases, once butyrate begins to be consumed. Dashed lines mark the time frame (days 18–55) during which butyrate was daily added to the media. (D) The frequency of different genotypes within the three populations to which butyrate was added between days 18 and 55. (E) The frequency of different genotypes within the three control populations to which butyrate was not added. (JPG) [file pgen.1010812.s008.jpg]

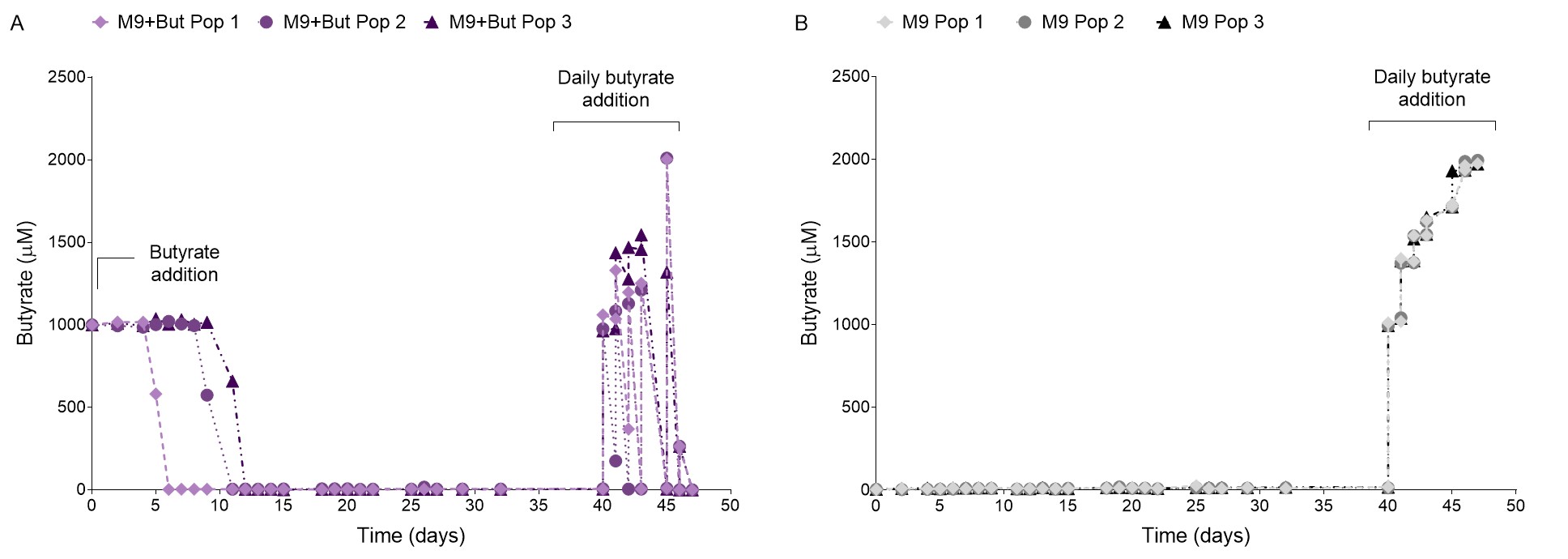

Supplement: S2 Fig — One day after initiation, cells from three LTSP experiments were filtered out of their LB media and re-inoculated into three flasks of M9 minimal media, either supplemented with 1 mM butyrate (A), or not (B). When butyrate was provided, it was initially consumed by day 12. Following four additional weeks, populations were sampled and assayed for their ability to consume butyrate, which was supplemented daily into the sampled populations. As shown, only populations that initially received butyrate (A) could then consume it, during the week following its addition into their media (arrows represent time points in which butyrate was added). (JPG) [file pgen.1010812.s009.jpg]

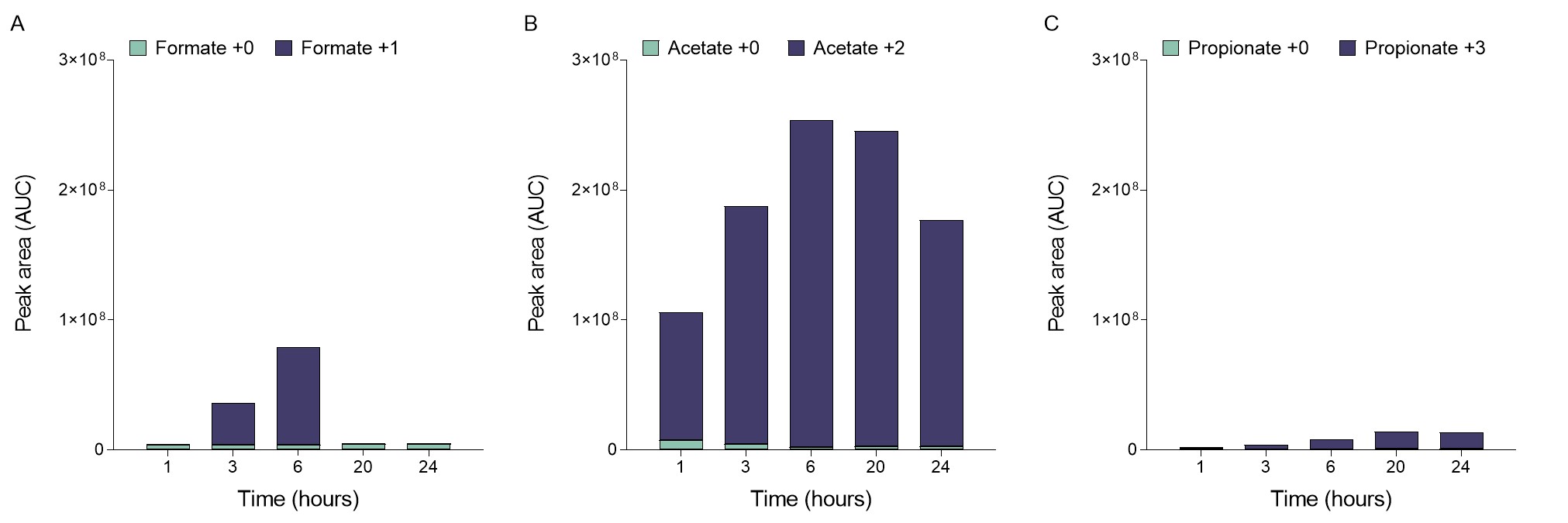

Supplement: S3 Fig — Full labeled glucose (M+6) was added into the M9 minimal media culture. Ancestral E. coli K12 MG1655 cells were sampled and assayed for their ability to produce butyrate during 24 hours of culture. As shown, only the SCFA formate (A), acetate (B) and propionate (C) were detected, in their unlabeled (M+0) and fully labeled (M+1, M+2, M+3, respectively) form. Butyrate, labeled or unlabeled, was not detected at any time point. These results indicate that E. coli does not produce butyrate via the acetyl-CoA (CoA) pathway. (JPG) [file pgen.1010812.s010.jpg]
